# Editorial: Artificial intelligence-based computer-aided diagnosis applications for brain disorders from medical imaging data, volume II

**DOI:** 10.3389/fnins.2023.1241926

**Published:** 2023-07-12

**Authors:** Fahmi Khalifa, Ahmed Shalaby, Ahmed Soliman, Safa Elaskary, Ahmed Refaey, Mohamed Abdelazim

**Affiliations:** ^1^Electrical and Computer Engineering, Morgan State University, Baltimore, MD, United States; ^2^Lyda Hill Department of Bioinformatics, Southwestern Medical Center, University of Texas, Dallas, TX, United States; ^3^Department of Computer Engineering, Faculty of Engineering, Mansoura University, Mansoura, Egypt; ^4^Department of Biomedical Equipment and Technology, Applied Health Sciences, Pharos University, Alexandria, Egypt; ^5^School of Engineering and Physical Sciences, University of Guelph, Guelph, ON, Canada; ^6^Electronics and Communications Engineering, Mansoura University, Mansoura, Egypt

**Keywords:** artificial intelligence, brain disorders, Electroencephalography (EEG), Alzheimer's disease, convolutional neural network (CNN), schizophrenia, multiple sclerosis, acute ischaemic stroke (AIS)

Artificial intelligence/Machine learning (ML) has recently received growing interest used to study of the etiology and mechanisms of several brain disorders by analyzing neuroimages and/or neuro signals, such as structural magnetic resonance imaging (MRI), functional MRI, and positron emission tomography (PET), Electroencephalography (EEG) signals, etc. Recent research work has achieved remarkable performance improvements to develop computer-aided diagnosis of many brain disorders. The latter include, autism, multiple sclerosis (MS), dementia, Alzheimer's disease (AD), gliomas, Schizophrenia, and Epilepsy.

Generally, manually processing of brain images/data is subjective, often time consuming, and chances of errors in the interpretation are not irrelevant (Segato et al., 2020). Thus, AI-based systems are growing fields for brain disorder analysis due to its capability to facilitate analyzing complex brain medical data to extract meaningful relationships in datasets, and reliable diagnostic cues for several clinical aims. For example, this can be used to eventually help physicians for more appropriate and precision medicine treatments.

Literature work utilizes variety of traditional ML or advanced end-to-end DL techniques to build CAD systems for brain data analysis. Both traditional and DL-based methods have shown significant performance in many brain applications. One of the limitations of traditional methods is, however; is that most of the learning techniques are often tailored to a specific application and usually need a lot of tuning and. On the other hand, CAD systems exploiting DL combined with recent progress in neuroimaging technologies have demonstrated unprecedented performance. to predict or provide better diagnosis of brain diseases. However, they require long training time and most of the techniques are less interpretable, i.e., lack explanation explanations.

This Research Topic is a continuation of the first topic (Shalaby et al., 2023) focusing on recent scientific findings of research work utilizing AI/ML/DL methods for neuroimaging-based brain disorder analysis. The target audience for this topic includes engineering and medical researchers/professionals with the purpose to stimulate discussion on the AI system designs for brain disorders for disease diagnostic and prognostic. The topic consists of 10 accepted articles that deal with AI/ML algorithms to investigate different problems. A brief summary of each article is provided below.

A study by Tan et al. is conducted to investigate the cognitive impairment relation with type 2 diabetes mellitus (T2DM) in an effort to assist clinicians analyze and predict cognitive impairment early. The method used a 3D 11-layer convolutional neural network (CNN) and documented that he classifier could identify T2DM-related cognitive decline with a classification accuracy of 84.85% and achieved an area under the curve of 92.65%. The study concluded the importance of the study to objectively analyze and predict patients' cognitive impairment, which can provide treatments to the patients in the early stage. It is important to block or delay the development of dementia. Despite the demonstrated performance, the study had a small sample size, which necessitate using larger sample sizes in the future studies. Also, both functional and structure images should be used for better diagnosis and strong evidence of the reported conclusions.

Alzheimer's disease (AD) is an acute degenerative disease affecting the elderly population all over the world. Although the development of AD is irreversible, preventive measures in the presymptomatic stage of AD can effectively slow down deterioration. Additionally, the detection of disease at an early stage crucial to the clinical treatment. Khan et al. introduced as study that utilized transfer learning (TL) to classify various stages of AD using MRI images. The architecture is exploited the pre-trained VGG16 and 19 architectures with addition of a layer-wise transfer learning. Their model can distinguish between four groups normal controls and various stages of AD. The latter is stratified as early mild cognitive impairment (EMCI), late mild cognitive impairment (LMCI), and AD. Conducted experimental results demonstrated the superiority of the proposed approach. Additionally, the comparison with existing methods demonstrates that the proposed framework is superior in terms of accuracy and prediction. We achieved an accuracy of 97.89% for the proposed 4-way classification. The main limitation of the study is the number of images used for system evaluation and that the TL-based architecture requires resizing of the input images, which results in loss of texture information when scaling down.

Unlike Khan et al. and Duan et al. exploited fluorodeoxyglucose positron emission tomography (FDG-PET) in an AD study using ML. The study hypothesized the detection of the metabolism of glucose in patients' brains can help to identify changes related to AD before brain damage occurs. The authors introduced a broad network-based model to enhance the features of FDG-PET extracted via 2D CNN for early diagnosis of AD (BLADNet) through PET brain. BLADNet can search for information over a broad space through the addition of new BLS blocks without retraining of the whole network, thus improving the accuracy of AD classification. Experiments were conducted on a dataset containing 2,298 FDG-PET images of 1,045 subjects from the ADNI database and the reported results demonstrate the superiority of the BLADNet in EMCI and LMCI classification to those used in previous studies on early diagnosis of AD with FDG-PET. The reported results are encouraging and the model can be integrated into the clinical workflow as a powerful auxiliary diagnosis tool for reading PET imaging of patients with AD. However, the study has some limitations including the fact that many patients may have neurological diseases other than AD that can affect the prediction results. The model could not provide interpretable information for radiologists of patients gradually progressing from MCI to AD, despite that t-SNE dimension reduction documented that. Thus, validation in large cohort and a more general patient population should be conducted as the actual clinical prediction is much more complicated.

An ML-based pipeline is introduced by Ricciardi et al. in a retrospective study using quantitative MRI-derived features to correlate with multiple sclerosis (MS), severity. The authors produced a multi-modal set of quantitative MRI features encompassing relaxometry, microstructure, sodium ion concentration, and tissue volumetry. In an effort to use them improve the understanding of the underlying disease by better delineating its alteration patterns. Classical ML classifier, namely random forest (RF) was employed to discriminate between HC, CIS, relapsing remitting (RR) and secondary progressive (SP) MS patients. Additional evaluation for each classification task was dedicated to identifying the relative contribution of each MRI-derived tissue property to the classification task itself. The reported binary accuracies [99% (HC vs. SP) and 95% (CIS vs. SP); 82 (HC vs. RR) and 83% (CIS vs. RR); 76% (RR vs. SP), and 79% (HC vs. CIS)] offered specific insights for different classification tasks, highlighting the need for more research emphasizing the role of quantitative MRI in the lesions of early CIS and MS subjects to score risks of progression. Similar to Tan et al., the sample sized used in the evaluation is small (e.g., SP group has only 13 subjects) which also introduce bias in the classifier. Despite the study showed that different MRI features appear to be biophysically meaningful when discriminating CIS and clinically defined MS phenotypes, the authors emphasized on additional studies with larger sample size and histological evidence are required to substantiate these findings.

In as study for acute ischaemic stroke (AIS), Xu et al. investigated the potential of medical images-derived features to predict the clinical outcome of patients with AIS. Particularly, this retrospective study investigated and validate a radiomics nomogram for predicting AIS prognosis using diffusion-weighted imaging (DWI) and fluid-attenuated inversion recovery (FLAIR) images. The study cohort included 257 patients from three clinical centers. Their framework extracted radiomic features from radiologist-defined regions of interest (ROIs) using ITK-SNAP^1^ software. Then, two feature selection methods, minimum redundancy and maximum (mRMR) and the least absolute shrinkage and selection operator algorithm (LASSO) are used to select the candidate features. Experiments based on extracted radiomics either DWI or FLAIR, as well as DWI-FLAIR, were established. A radiomics nomogram with patient characteristics and radiomics signature was built using a multivariate logistic regression model. The presented quantitative results showed that FLAIR-derived radiomics have a similar performance with the DWI- DWI-FLAIR-derived radiomics–prediction performance AUCs of 0.922 (95% CI: 0.876–0.968), 0.875 (95% CI: 0.815–0.935), and 0.895 (95% CI: 0.840–0.950), respectively. The results document the ability of the non-invasive clinical-FLIAR radiomics nomogram in predicting ASI prognosis after thrombolysis, as step that could help clinicians plan rehabilitation for stroke patients. Despite the study demonstrated good calibration, and decision curve analysis confirmed the clinical value of this nomogram, the cohort sample size is relatively small, which may lead to overfitting, similar to Tan et al. and Ricciardi et al.. This had been compensated in part by that the study used patient data with different MRI scanners and other two clinical centers as two external validation sets. In addition, patients with lacunar infarction and posterior circulation stroke were excluded from the current study, which was justified for the need to ensure strict grouping and no bias in the radiomics results.

In literature, a few studies have explored the differences in functional connectivity (FC) between the different Schizophrenia by analyzing gray matter volume (GMV), which is a challenge research area due to the fact that the characteristics of brain networks are still unknown. Zhu et al. aimed in their work to study the alterations of FC between deficient schizophrenia (DS) and non-deficient schizophrenia (NDS) based on the ROI obtained by ML algorithms and differential GMV. They investigated and analyzed the relationships between the alterations and the clinical symptoms were analyzed. In addition, the thalamic FC imbalance in the two groups was further explored using resting-state fMRI scans. Investigation on a cohort of 85 subjects (16 DS, 31 NDS, and 38 HC) was conducted based on GMV image data and support vector machine (SVM) to classify DS and NDS. Clinical scales including the Brief Psychiatric Rating Scale (BPRS), the Scale for the Assessment of Negative Symptoms (SANS), and the Scale for the Assessment of Positive Symptoms (SAPS) were also used for further evaluation. Brain regions with high weight in the classification were used as seed points in whole-brain FC analysis and thalamic FC imbalance analysis. Finally, partial correlation analysis explored the relationships between altered FC and clinical scale in the two subtypes. The results obtained a relatively high SVM-based classification accuracies and authors concluded that the ROI from GMV highlighted the difference in FC between DS and NDS from the local to the brain network, which provides new information for exploring the neural physiopathology of the two subtypes of schizophrenic. The findings of this study corroborate the previous conclusion of the hypothesis that thalamocortical imbalance in both of the two subtypes of schizophrenia. In addition, the FC value of THA.R and SMN in the DS group was negatively correlated with the SANS score, but the FC value of THA.R and SMN in the NDS group was positively correlated with the SAPS score, which deepens the understanding of the pathological mechanism of the two subtypes of schizophrenia. However, all the reported results utilized only SVM-based ML with a small imbalanced groups and larger samples in the future are needed to confirm current findings. Finally, the study only included the right thalamus in the study of thalamocortical imbalance, which will make the attribution of thalamic FC imbalance analysis in the two subtypes of schizophrenia incomplete.

Li et al., proposed deep learning method for objective assessment of chronic primary pain using the EEG and PLI matrices and frequency band data of auditory stimulation state. The aim was to distinguish among chronic primary pain (CPP), depressive disorder (DD), and HC subjects. The study was conducted on a cohort 67 subjects (23 healthy subjects, 22 patients with depression, and 22 patients with CPP) under the auditory oddball paradigm. The results showed that the CNN classification model of EEG is better than that of PLI, with the highest accuracy of 85.01% in Gamma band in former and 79.64% in Theta band in later. The study concluded that the current approach may be a step toward an EEG-based auxiliary diagnosis of CPP. Namely, the authors suggest that the EEG of each frequency band may play a role in the pathophysiology of CPP. Third, the abnormal pattern of EEG activity in CPP patients may represent a potential new therapeutic target, such as intervention through non-invasive brain stimulation technology or neural feedback methods targeting Theta and Gamma oscillation and FPN networks. In particular, the emerging transcranial magnetic stimulation can modulate the oscillation and synchronization of neurons at specific frequencies, so it may be a promising method for pain modulation (Polanía et al., 2018). This study has several limitations that should be considered. Firstly, the sample size was relatively small with only 67 subjects, limiting the generalizability of the findings. A larger and more diverse sample would increase the reliability and applicability of the method. Secondly, the study focused on a specific experimental condition and population, using EEG and PLI data collected under the auditory oddball paradigm. It is important to validate the results in different conditions and on a broader range of subjects to ensure the method's generalizability. Lastly, the choice of specific features, such as EEG and PLI matrices and frequency band data, may introduce biases and overlook other relevant information.

A similar study to Li et al. for major depressive disorder (MDD) was conducted by Zhang et al. to investigate common and unique patterns of homotopic connectivity and their relationships with clinical characteristics in patients with MDD. Experiments were conducted on balanced cohort (42 MDD patients and 42 HCs), where a range of clinical variables were collected for group comparisons using correlation analysis, SVM, and voxel-mirrored homotopic connectivity (VMHC). In particular, exploratory eye movement (EEM), event-related potentials (ERPs) and resting-state functional magnetic resonance imaging (rs-fMRI) data were analyzed. The study showed that patients with MDD showed decreased VMHC in the insula, and increased VMHC in the cerebellum 8/vermis 8/vermis 9 and superior/middle occipital gyrus. SVM analysis using VMHC values in the cerebellum 8/vermis 8/vermis 9 and insula, or VMHC values in the superior/middle occipital gyrus and insula as inputs can distinguish HCs and patients with MDD with high accuracy, sensitivity, and specificity. The study conclusion was that decreased VMHC in the insula and increased VMHC values in the sensory-motor networks may be a distinctive neurobiological feature for patients with MDD, which could potentially serve as imaging markers to discriminate HCs and patients with MDD. In total, there are still some limitations in this study. Clearly, this is a small preliminary report that needs to be replicated. Second, the study focused on patients who did not take medicine at the time of the first episode, and patients who had relapsed and did not take medicine for at least 2 weeks. The dissemination of research findings may be constrained for patients who experience relapses since the impact of antidepressant medications and number of episodes on brain spontaneous activity cannot be entirely ruled out. Also, the patients were scanned at baseline only and no investigation of neuronal activity after treatment were done.

An integrative prediction model, constructed based on radiomics of multiparametric MRI and clinical parameters was proposed by Wang et al. for predicting Sonic Hedgehog (SHH) and Group 4 (G4) molecular subtypes of pediatric medulloblastoma (MB). In their study, 7,045 radiomic features were extracted from T1-weighted (T1), contrast-enhanced T1 (T1c), T2-weighted imaging (T2), T2 fluid-attenuated inversion recovery imaging (T2FLAIR), and apparent diffusion coefficient (ADC) maps, using variance thresholding, SelectKBest, and Least Absolute Shrinkage and Selection Operator (LASSO) regression algorithms. Then, features were filtered (17 optimal features) using LASSO regression, and a logistic regression (LR) algorithm and Delong test was used to compare the differences between different models. The analysis was conducted on a retrospective preoperative MRI images and clinical data of 95 patients with MB, (47 and 48 cases of SHH and G4 subtypes, respectively). The overall testing accuracies based on the area under curve (AUC) of receiver operator characteristic (ROC) were 0.751 (95% CI: 0.587–0.915) and 0.849 (95% CI: 0.695–1.000), with and without clinical parameters, respectively (Delong's test with *p* = 0.0144). Decision curves and nomogram further validate that the combined model can achieve net benefits in clinical work and thus can potentially provide a non-invasive clinical approach to predict SHH and G4 molecular subtypes of MB preoperatively. Despite the promising results of the combined model, the study cohort has a small number of cases and the WNT and G3 groups were not included in the study. Further, the results of this study lack external validation to better assess the generalizability of the model.

A retrospective study by Bao et al. is conducted to assess/quantify hemodynamic parameters using CT angiography (CTA) in spontaneous vertebral artery dissection (sVAD). The authors investigated that if sVAD might tend to develop in vertebral artery hypoplasia (VAH) with hemodynamic dysfunction. The geometries of 14 patients (28 vessels) were reconstructed using Mimics and Geomagic Studio software from CTA. ANSYS ICEM and ANSYS FLUENT were utilized for mesh generation, set boundary conditions, solve governing equations, and perform numerical simulations. Slices were obtained at the upstream area, dissection or midstream area and downstream area of each VA. The blood flow patterns were visualized through instantaneous streamline and pressure at peak systole and late diastole. The hemodynamic parameters included pressure, velocity, time-averaged blood flow, time-averaged wall shear stress (TAWSS), oscillatory shear index (OSI), endothelial cell action potential (ECAP), relative residence time (RRT) and time-averaged nitric oxide production rate (TARNO). The study results showed a significant focal increased velocity in the dissection area of steno-occlusive sVAD with VAH compared to other nondissected areas (0.910 m/s vs. 0.449 vs. 0.566, p < 0.001). Also, focal slow flow velocity was observed in the dissection area of aneurysmal dilatative sVAD with VAH according to velocity streamlines. Based on the rsults, the authors concluded that steno-occlusive sVAD with VAH patients had abnormal blood flow patterns of focal increased velocity, low time-averaged blood flow, low TAWSS, high OSI, high ECAP, high RRT and decreased TARNO. These results provide a good basis for further investigation of sVAD hemodynamics and support the applicability of the CFD method in testing the hemodynamic hypothesis of sVAD. In summary, this study has several limitations that should be considered. Firstly, it had a retrospective design, which relies on existing data and may be subject to bias or incomplete information. Prospective studies would offer more robust data collection. Secondly, the study had a small sample size of only 14 patients, which limits the statistical power and generalizability of the findings. Additionally, the numerical simulations performed using ANSYS ICEM and ANSYS FLUENT are subject to certain assumptions and limitations. The accuracy of the results depends on the accuracy of the models and boundary conditions used in the simulations.

In total, the collection comprises 10 comprehensive papers that tackle different but extremely relevant to AI-based CAD systems for brain disorders. The issue papers focus on development of better diagnostic tools to analyze medical images/signals from patients with various brain disorders. We extend our heartfelt gratitude to all the contributing authors who submitted their exceptional research work, as their contributions have enriched the content and breadth of this publication. Furthermore, the guest editors would also like to acknowledge the contribution of many experts who have participated in the review process to select candidate papers for publications by providing helpful and constructive feedback to the authors to improve the contents and presentations of their articles. Finally, we would like to extend our gratitude to the Editor-in-Chief and the publishing team for their unwavering support throughout the different stages of this Research Topic. Their valuable input, suggestions, comments, and feedback have been instrumental in ensuring the success of this endeavor.

In conclusion, we believe that this Research Topic will make a notable contribution to the scientific community by disseminating cutting-edge research and fostering collaboration among researchers and practitioners in the field of AI-based CAD systems for brain disorders.

## Author contributions

All authors have participated in preparing the Research Topic, managing the peer review of the submitted manuscripts, and contributing to the writing and review of the editorial.

## Footnotes

^1^ www.itksnap.org
